# Multimodality imaging of paragangliomas of the head and neck

**DOI:** 10.1186/s13244-019-0701-2

**Published:** 2019-03-04

**Authors:** Jarett Thelen, Alok A. Bhatt

**Affiliations:** 1 University of Rochester Medical Center, Rochester, USA; 2 Department of Imaging Sciences, University of Rochester Medical Center, 601 Elmwood Avenue, Rochester, NY 14607 USA

**Keywords:** Paraganglioma, Extra-adrenal, Glomus tumor, Neoplasms, Head, Neck

## Abstract

Paragangliomas arise from paraganglion cells which serve varied regulatory tasks in the body. When these cells demonstrate neoplasia within the head and neck, they typically present in characteristic locations including the carotid space, the jugular foramen, the middle ear, and along the course of the vagus nerve. The goal of this article is to review the relevant anatomy related to head and neck paragangliomas, as well as their typical imaging characteristics on cross-sectional imaging including CT, MR, ultrasound, and nuclear medicine studies. Additionally, differential considerations, as well as relevant involvement of adjacent structures which should be conveyed to the clinician, will be discussed.

## Key points

- Paragangliomas arise from paraganglion cells. These cells serve varied regulatory tasks in the body, including chemoreceptor functions, which allow the body to respond to stresses such as hypoxia, hypercapnia, and hypoglycemia.
- The imaging evaluation of head and neck paragangliomas utilizes multiple cross-sectional imaging modalities including CT, MR, ultrasound, and various nuclear medicine techniques.
- Head and neck paragangliomas most common anatomic locations include the carotid space, jugular foramen, middle ear, and along the course of the vagus nerve.
- Paragangliomas are vascular tumors and demonstrate avid enhancement on both CT and MR. They also often exhibit a characteristic “salt and pepper” appearance on MR.

## Introduction

Paragangliomas, also known as glomus tumors, arise from paraganglion cells which form the basis of the extra-adrenal neuroendocrine system. These cells serve varied regulatory tasks in the body, including chemoreceptor functions, which allow the body to respond to stresses such as hypoxia, hypercapnia, and hypoglycemia. When these cells demonstrate neoplasia within the head and neck, they typically present in characteristic locations, and with common symptomatology; thus, understanding these key features in conjunction with their imaging appearance can lead to accurate and prompt diagnosis. The imaging evaluation of head and neck paragangliomas utilizes multiple cross-sectional imaging modalities to evaluate for extent of disease and to help guide surgical management by evaluating for osseous and neurovascular involvement. In this article, we will review the expected anatomic locations of paragangliomas and their general imaging appearance on computed tomography (CT) and magnetic resonance (MR), as well as CT and MR angiography. Additionally, we will discuss other differential considerations for lesions found in these locations. By understanding these fundamental concepts, the radiologist will be able to accurately differentiate a generally benign paraganglioma from a potentially more aggressive head and neck neoplasm.

**Fig. 1:**
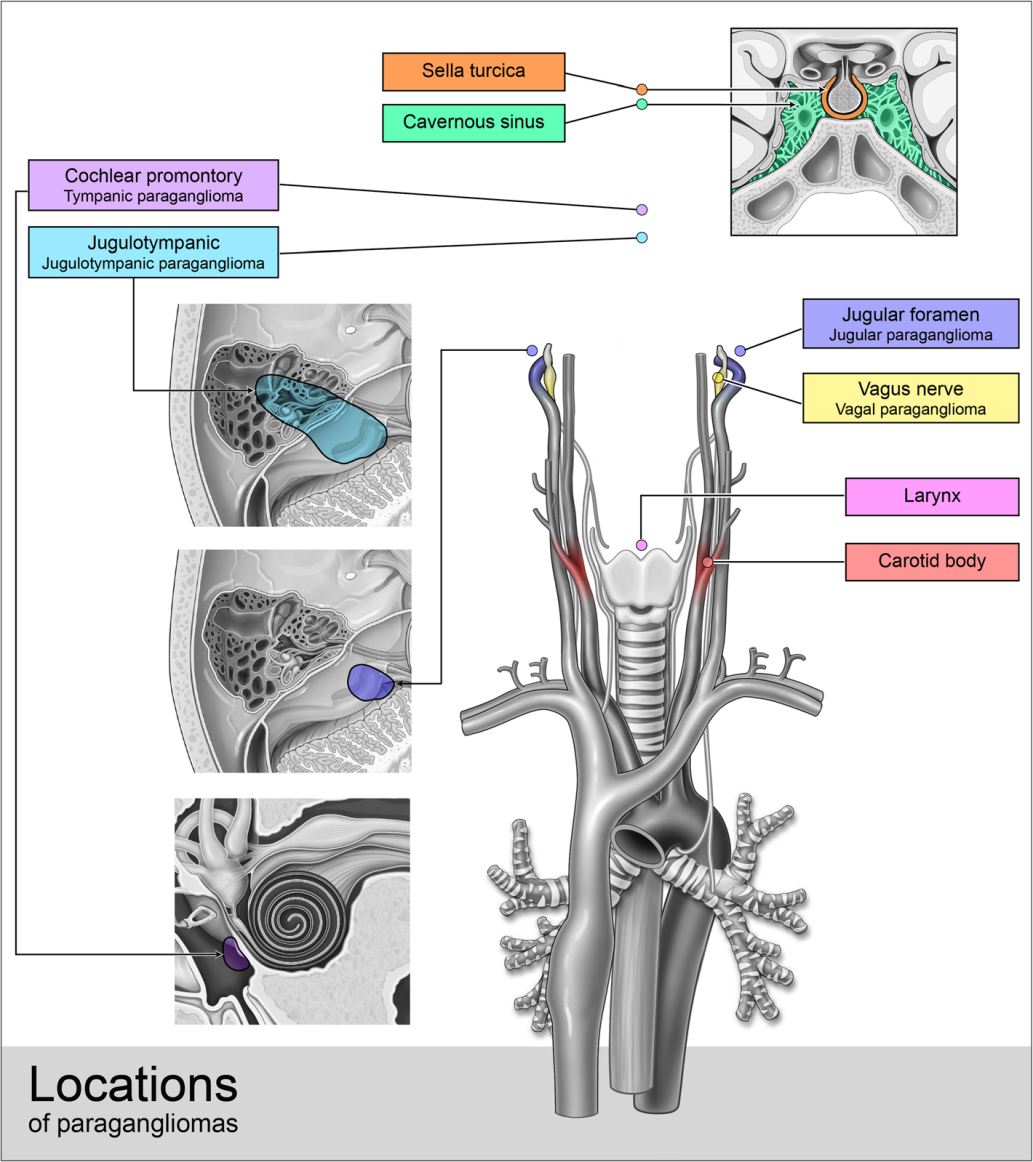
Anatomic regions of head and neck paragangliomas

Their most common anatomic locations include the carotid space, jugular foramen, middle ear, and along the course of the vagus nerve (Fig. 1). The majority of head and neck paragangliomas are benign and only locally invasive, with only approximately 2–13% of paragangliomas demonstrating malignancy [1]. Malignancy is defined by the anatomic presence of metastasis, as there are no current histopathologic diagnostic criteria which can accurately define malignant paragangliomas from their benign counterparts [2]. Malignant paragangliomas of the head and neck most commonly demonstrate regional nodal metastasis, and distant metastasis is extremely uncommon [3].

While the majority of cases are sporadic, approximately 40% are thought to be hereditary [4]. These familial tumors tend to occur earlier than their sporadic counterparts, with a peak prevalence of 30–35 years of age [5]. Additionally, familial paragangliomas are more commonly multicentric than sporadic tumors. Paragangliomas of the head and neck are more common in females, with this predilection most common in jugular (3:1) and tympanic subtypes (6:1) [6]. Paragangliomas are associated with multiple syndromes and genes and are commonly seen in patients with von Hippel-Lindau (VHL), neurofibromatosis type I (NF I), and multiple endocrine neoplasia type II (MEN II) [7, 8].

### Imaging characteristics

**Fig. 2:**
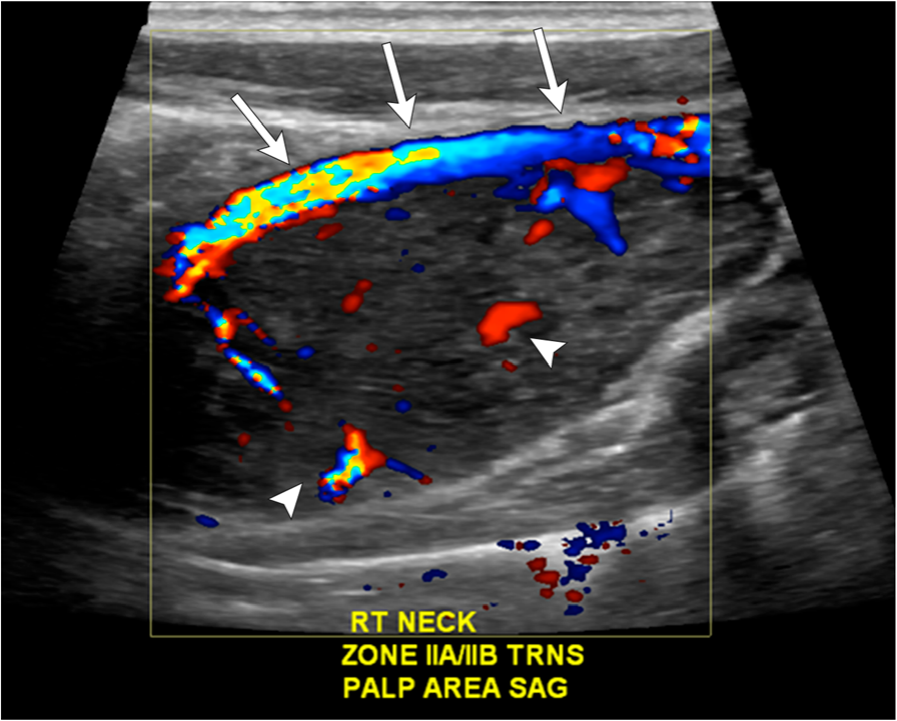
Color Doppler ultrasound image of the right cervical neck demonstrates a relatively hypoechoic, well-circumscribed mass with internal vascular flow (white arrows) in the right level II region. The mass splays the external carotid artery superficially (arrowheads)

Ultrasound is typically utilized early in the diagnostic process, often for initial evaluation of a palpable neck mass. Sonographic evaluation of paragangliomas demonstrates a well-defined, heterogeneously hypoechoic mass, with marked internal vascularity on color Doppler (Fig. 2). Careful assessment of the displacement pattern of the internal and external carotid arteries can raise the suspicion for carotid body and vagal paragangliomas and prompt cross-sectional imaging evaluation.

Magnetic resonance (MR) imaging is the most sensitive imaging modality for evaluation of paragangliomas given its superior soft tissue resolution, and it can readily characterize these tumors from other head and neck neoplasms. Compared to the adjacent soft tissues, paragangliomas typically demonstrate hypointense signal on T1-weighted sequences and isointense to hyperintense signal on T2-weighted sequences. Given the vascular nature of most paragangliomas, internal flow voids are commonly seen, particularly on T2-weighted sequences. More rarely, areas of hyperintense intertumoral hemorrhage can be seen on both T1- and T2-weighted sequences. Interspersing of these, hypointense flow voids and hyperintense areas of hemorrhage results in a characteristic “salt and pepper” appearance which is most apparent in tumors greater than 1 cm. Paragangliomas most commonly demonstrate avid, homogenous enhancement after administration of intravenous gadolinium contrast agents.

On computed tomography (CT), paragangliomas present as a well-defined soft tissue attenuation mass. They most commonly demonstrate homogenous, avid enhancement after administration of intravenous contrast, though heterogeneity can occur in lesions with intratumoral thrombosis or hemorrhage. Additionally, paragangliomas may cause local erosion of adjacent bony structures, and CT imaging is excellent for evaluation of osseous involvement. In the case of paragangliomas involving the skull base, dedicated high-resolution temporal bone CT is typically performed to evaluate the involvement of key structures and to guide surgical management.

Angiography, either with CT angiography (CTA), MR angiography (MRA), or digital subtraction angiography (DSA), is typically performed either as an adjunct to CT or MR, as well as in the preoperative setting [9]. These modalities allow for evaluation of tumor perfusion and identification of feeding vessels, which can guide subsequent embolization or surgical approaches [10]. Given the vascular nature of paragangliomas within the head and neck, angiography typically demonstrates multiple enhancing, feeding peri-tumoral vessels, including arterial and venous vasculature. Additionally, both CTA and MRA are useful for demonstrating the presence of multicentric disease [11, 12].

**Fig. 3:**
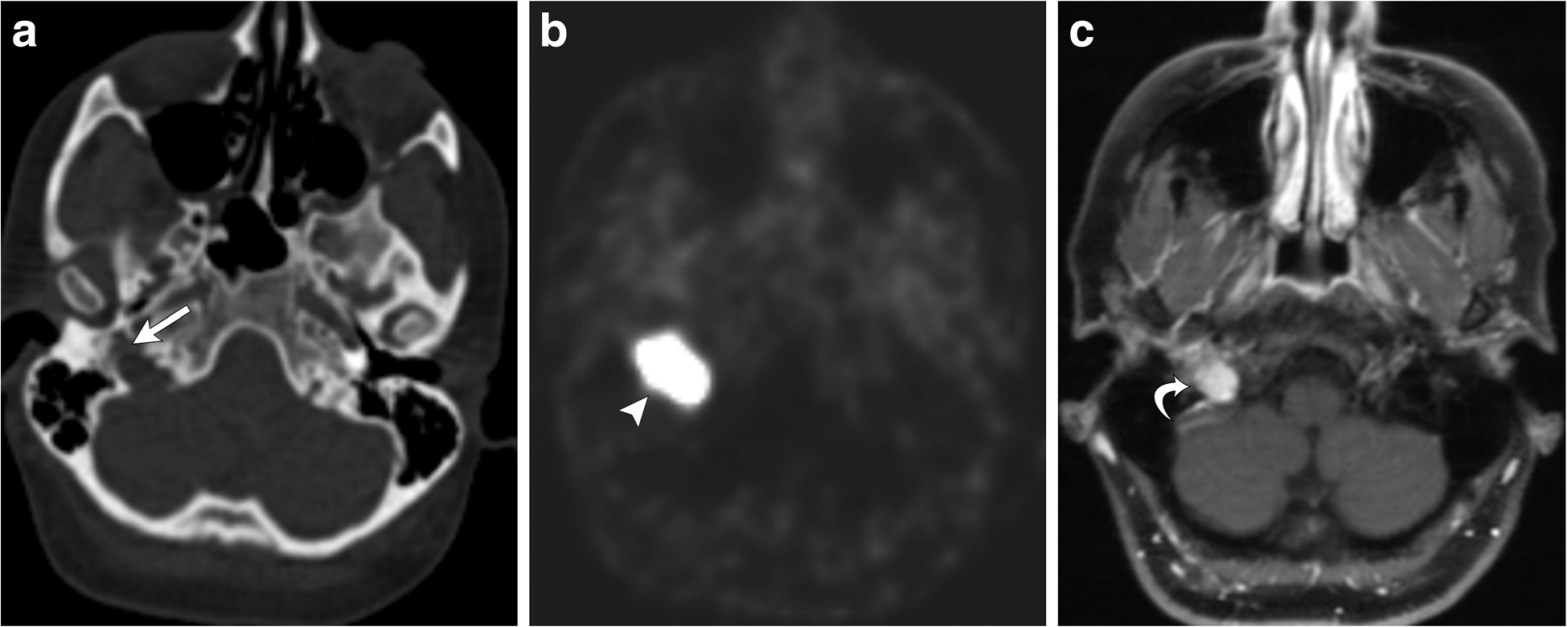
Axial CT (**a**) demonstrates widening right jugular bulb, with a moth-eaten appearance of the walls of the right jugular foramen (white arrow). Axial attenuation-corrected PET (**b**) demonstrates marked FDG uptake within the jugular foramen consistent with hypermetabolic activity (white arrowhead). T1-weighted post-contrast MR (**c**) demonstrates a well-defined, enhancing, expansile mass centered in the right jugular foramen (curved white arrow)

Various nuclear medicine imaging techniques may be used to evaluate for multicentric or metastatic disease, including I-131 and I-123 metaiodobenzylguanidine (MIBG), In 111 octreotide, and F-18 PET/CT [13, 14], demonstrating focally increased uptake within the lesion (Fig. 3.

### Carotid body paragangliomas

Carotid body paragangliomas are the most common paragangliomas within the head and neck, accounting for approximately 60% of all cases. The majority of cases occur in older patients, ranging from 40 to 70 years of age with a median age of 57 [15]. As their name suggests, they arise from the carotid body, a grouping of chemoreceptors which reside between the internal and external carotid arteries at the bifurcation of the common carotid artery. The carotid body provides regulatory function for both oxygenation, carbon dioxide, and pH within the blood. Carotid body tumors classically present as slow-growing, painless swelling in the lateral neck [16], often laterally mobile but fixed vertically (known as Fontaine’s sign). As the tumor grows within the carotid space, it can compress the adjacent nerves (most commonly the vagus nerve), resulting in symptoms such as dysphagia, hoarseness, or Horner’s syndrome [17]. The majority of carotid body paragangliomas are unilateral, though bilateral lesions are seen in approximately 18% of patients [6]. Increased incidence (up to 10% of patients) of sporadic carotid body paragangliomas is seen in patients living at high altitude, or in the setting of chronic obstructive lung disease [18].

**Fig. 4:**
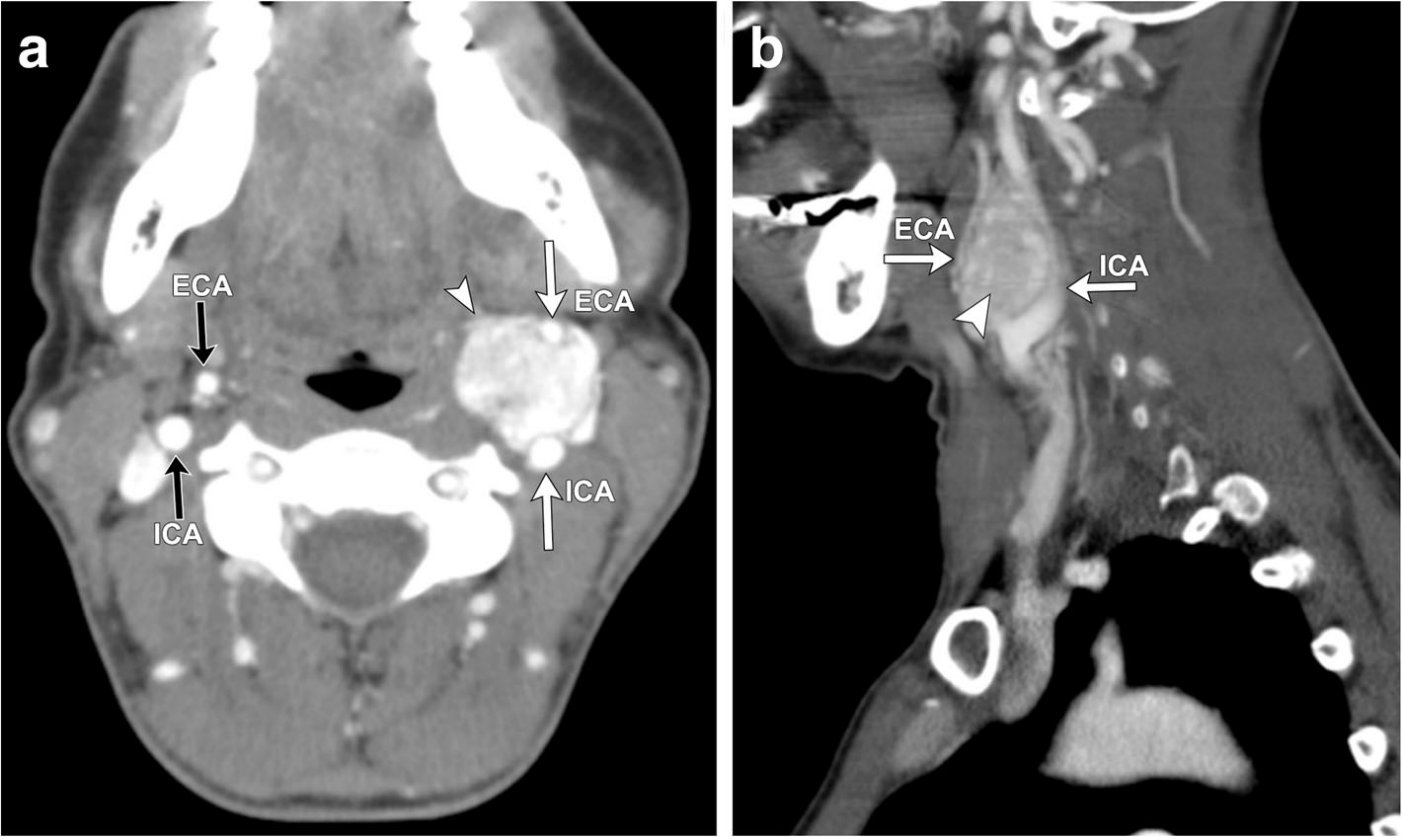
Axial (**a)** and sagittal (**b**) contrast-enhanced CT of the neck demonstrates a well-defined, avidly enhancing soft tissue mass (white arrowheads) within the left carotid space. The mass splays the proximal external and internal carotid arteries (white arrows) at the level of the carotid bifurcation, characteristic of a carotid body tumor (as opposed to a glomus vagale, which typically displaces the ICA and ECA together). The mass encases less than 50% of the left ICA. Note the normal relation of the right ECA and ICA on the contralateral side of the neck (black arrows)

**Fig. 5:**
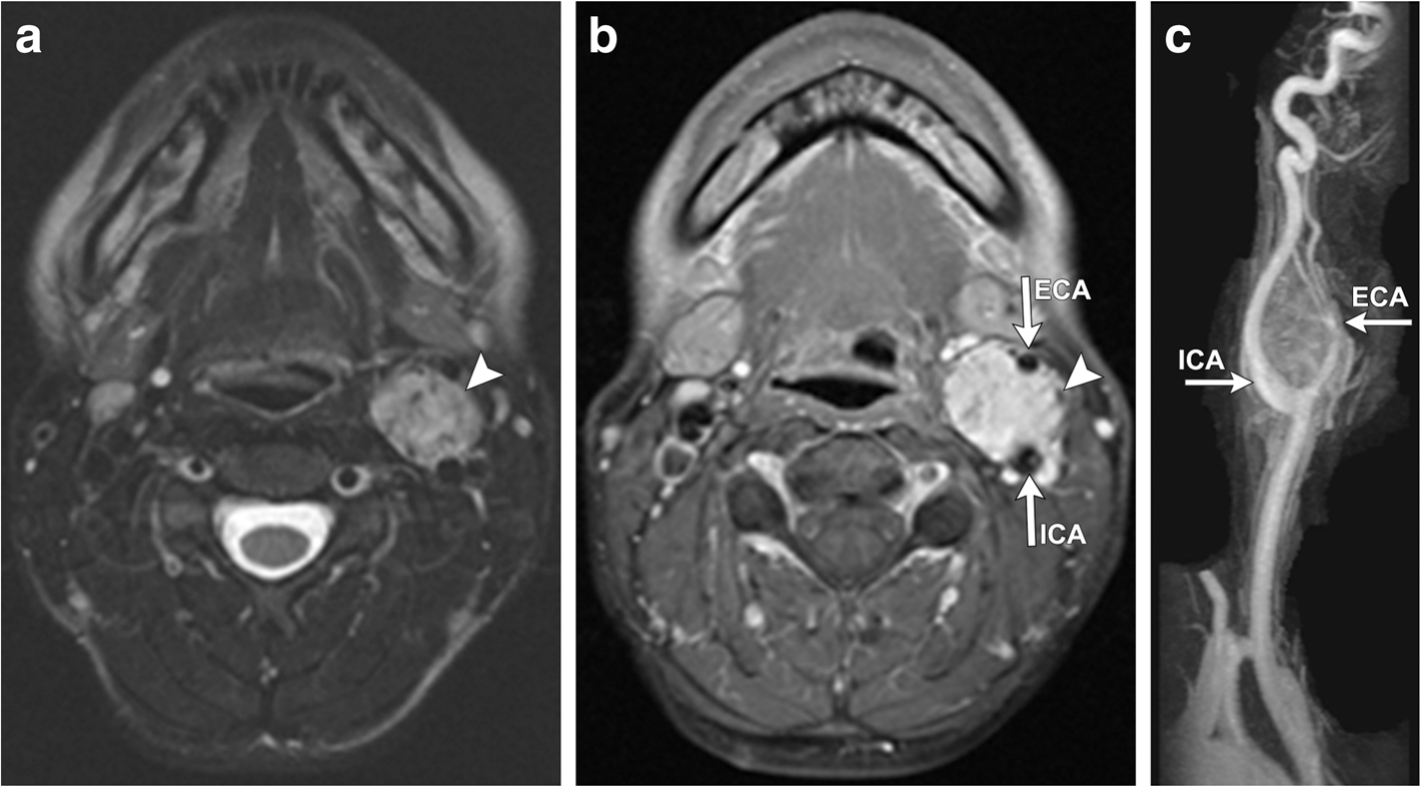
**a** Axial T2-weighted FSE MR images of the neck again demonstrate a well-defined, high signal soft tissue mass (white arrowhead) within the left carotid space, with multiple punctate foci of low signal, representing vascular flow voids. Punctate areas of hyperintensity (hemorrhage) as well as these flow voids produce a classic “salt and pepper” appearance. **b** Axial T1 post-contrast images demonstrate avid enhancement of the mass (white arrowhead), again splaying the proximal external and internal carotid arteries (white arrows). **c** 3D maximum intensity projection (MIP) time of flight angiography demonstrates a highly vascular tumor situated within the carotid bifurcation, with numerous enhancing feeding vessels branching from the adjacent ICA and ECA (white arrows)

**Fig. 6:**
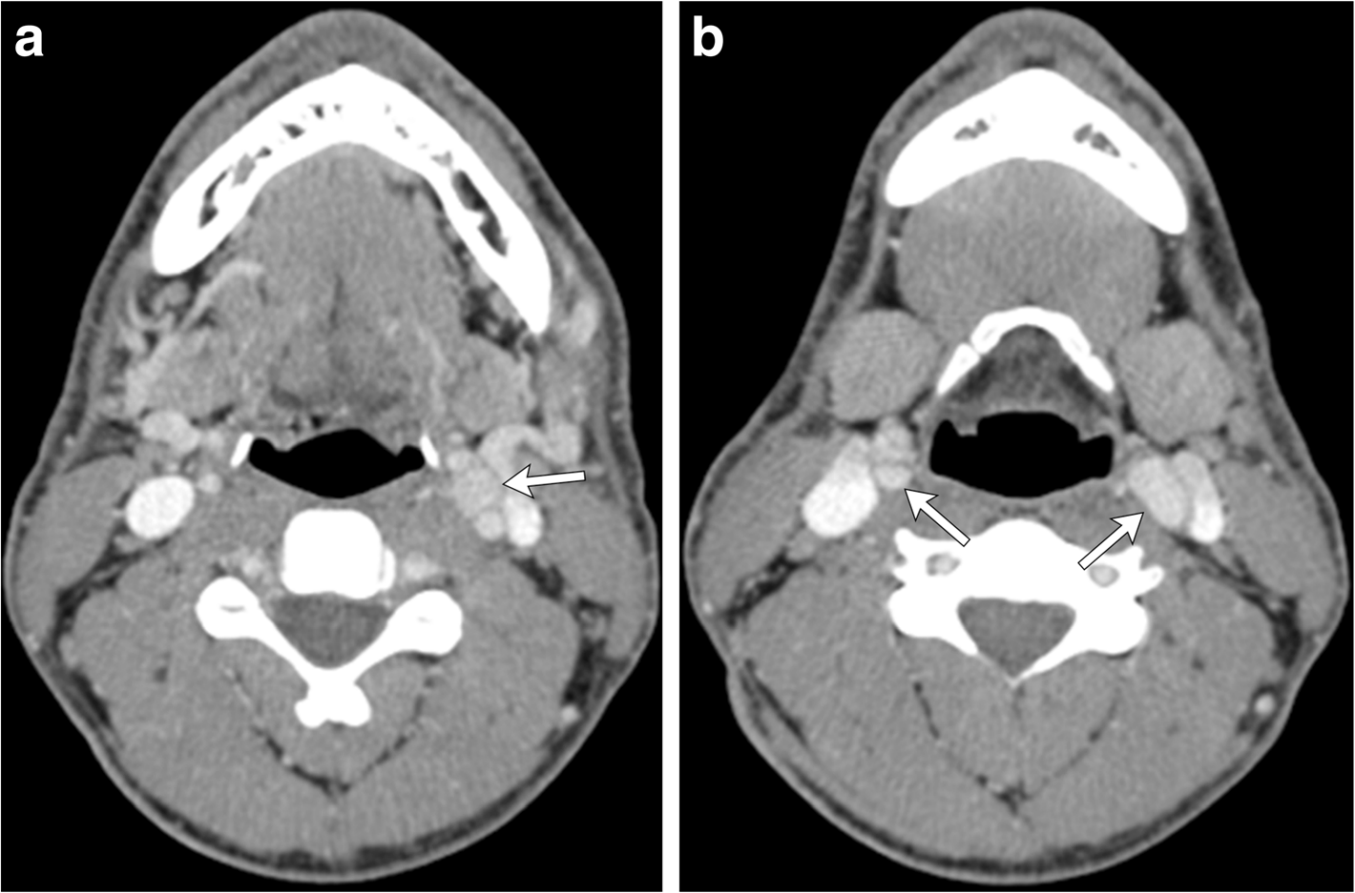
Axial contrast-enhanced CT images of the neck through superior (**a**) and inferior (**b**) portions of the carotid bifurcations demonstrate well-defined, avidly enhancing soft tissue masses (white arrows) within the bilateral carotid spaces, which splays the external and internal carotid arteries. Bilateral carotid body paragangliomas are much more common in patient’s living at high altitude

**Fig. 7:**
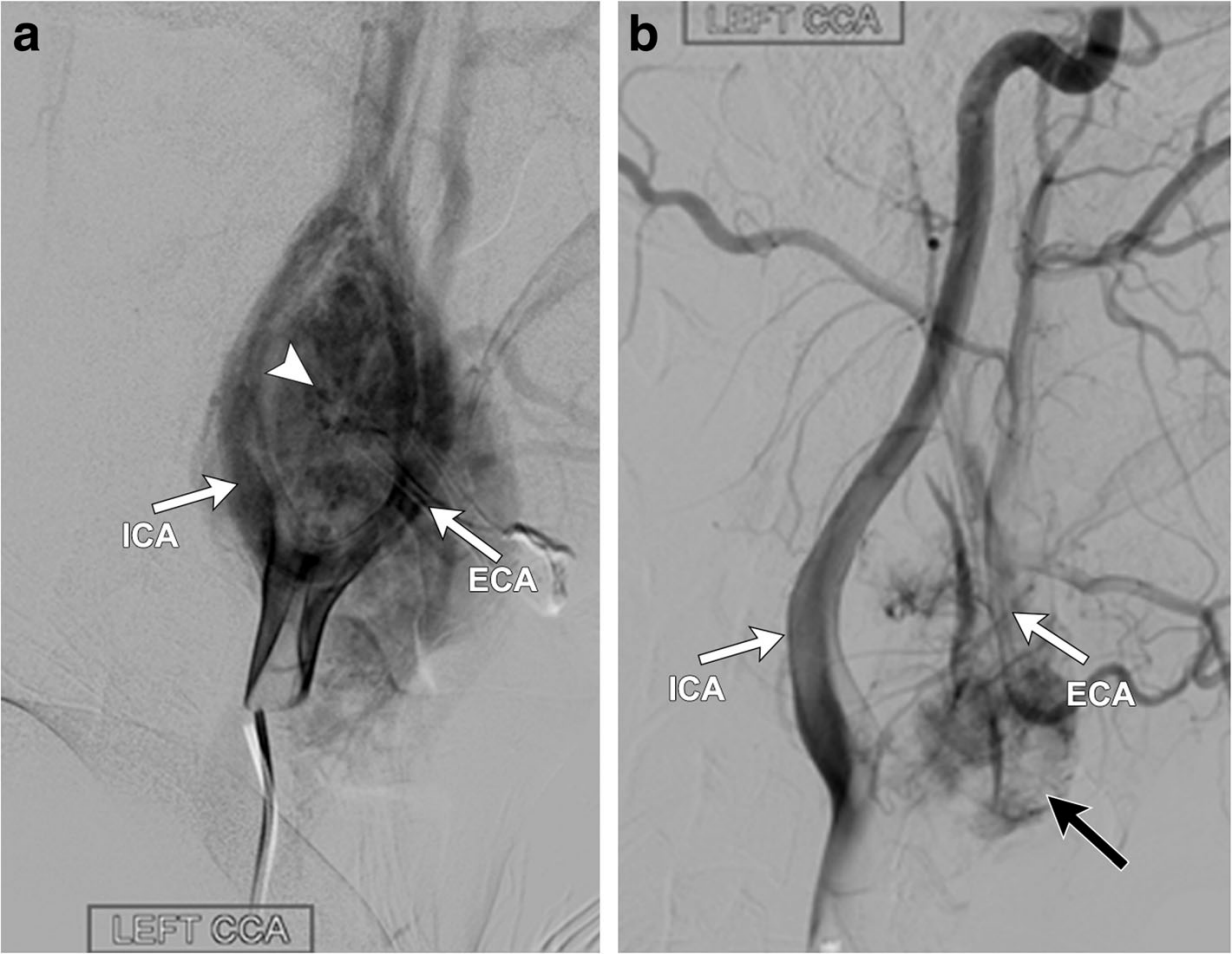
Patient subsequently underwent pre-surgical embolization of the mass. Conventional angiographic images (**a**) after selection of the left common carotid artery demonstrate an intense tumor blush (white arrowhead) arising between the left internal and external carotid arteries (white arrows). Subsequent images post particle embolization (**b**) of the tumor demonstrate marked interval decrease in tumor blush, with only a small residual portion of blush (black arrow) along the proximal ECA. Final pathology was consistent with a carotid body paraganglioma

Both CT and MR are useful for initial evaluation of potential carotid body paragangliomas and demonstrate an enhancing soft tissue attenuation mass situated within the carotid space centered at the carotid bifurcation. Associated mass effect classically results in splaying of the internal and external carotid arteries, resulting in the characteristic “lyre sign” [16], rather than displacing them together (Figs. 4, 5, 6, and 7). Differential considerations when encountering a mass in this location include schwannomas and neurofibromas; however, these tumors are generally less vascular and should not demonstrate the characteristic flow voids seen in paragangliomas. Additionally, hypervascular metastases such as those seen with primary renal or thyroid malignancy should be considered. When evaluating a carotid body tumor, it is important to describe the degree of vascular encasement, particularly of the ICA. By providing the degree of maximum circumferential contact of the ICA by the carotid body tumor, the lesion can be placed into the Shamblin classification system, which is used to predict vascular morbidity and cranial nerve deficit from resection [19].

### Vagal paragangliomas

Vagal paragangliomas occur within one of the ganglia of the vagus nerve (CN X). They are the least common head and neck paraganglioma, accounting for approximately 5% of all cases [20]. Vagal paragangliomas are typically seen in middle-aged or elderly patients, with a mean age of 48 years old [6]. Like carotid body tumors, they typically present as a slow-growing, painless neck mass. However, symptomatology differs in that both vagal nerve palsy and hoarseness due to vocal cord paralysis are quite common, seen in 33–37% of patients [6, 21].

**Fig. 8:**
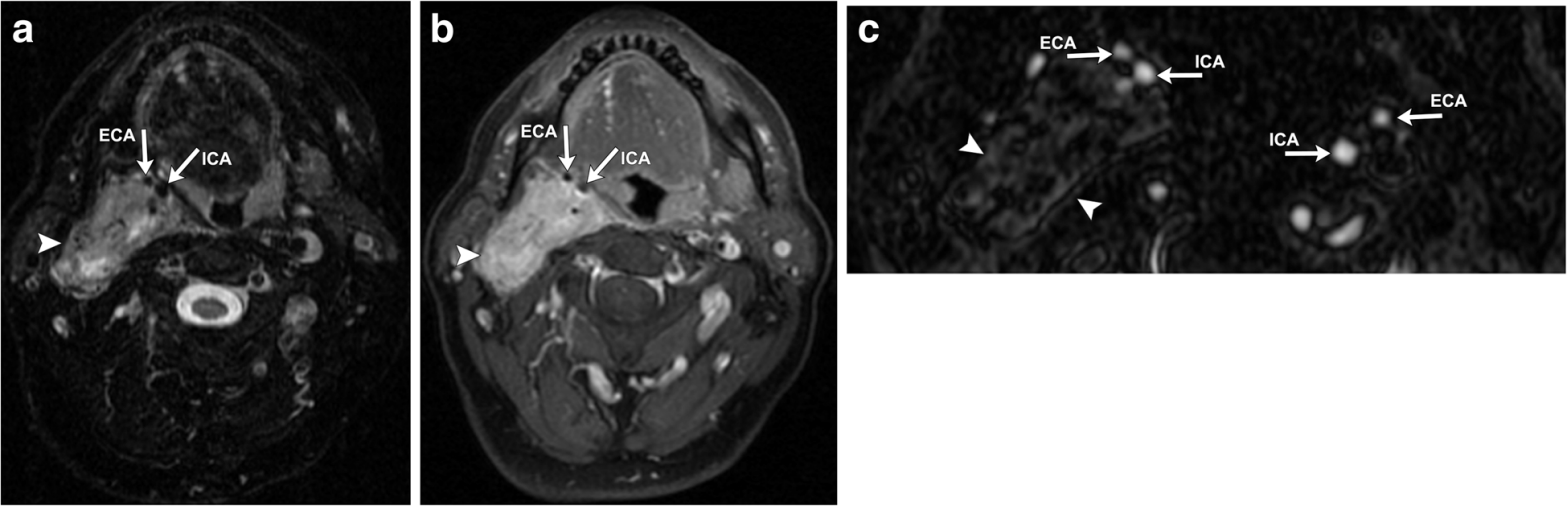
Axial T2 FSE (**a**) and T1 post-contrast (**b**) CT images demonstrate a large, well-defined expansile mass centered within the right carotid space (white arrowhead). The lesion is heterogeneously hyperintense on T2 and avidly enhances post-contrast. The flow voids of the right internal and external carotid arteries are displaced together anteromedially (white arrows). Axial 2D time-of-flight MR angiography (**c**) demonstrates the mass (white arrowheads), as well as clearly delineating its resultant mass effect exerted upon the right ICA and ECA, which are pushed together anteriorly within the neck (white arrows). This is in contrast to the contralateral carotid vessels on the left which are in normal position

Vagal paragangliomas can occur anywhere along the course of the vagus nerve but are typically seen high in the suprahyoid neck within the inferior (nodose) ganglion, which forms just as the vagus exits the jugular foramen [22]. They demonstrate similar imaging characteristics on CT and MR to carotid body tumors, but because of the posterolateral course of the vagus nerve with respect to the internal and external carotid arteries, its resultant mass effect causes these vessels to be displaced together anteromedially (Fig. 8), rather than splayed apart. CT is of particular importance when evaluating high vagal paragangliomas, as its superior osseous resolution can establish if there is osseous involvement of the anterior skull base.

### Jugular paragangliomas

Jugular paragangliomas are the second most common head and neck paraganglioma [6], and the most common tumor found within the jugular fossa. They arise from paraganglia associated with the adventitia of the jugular bulb. The average presentation is 53 years of age; the most common presenting clinical symptoms are unilateral tinnitus or hearing loss (51% of patients) [6].

**Fig. 9:**
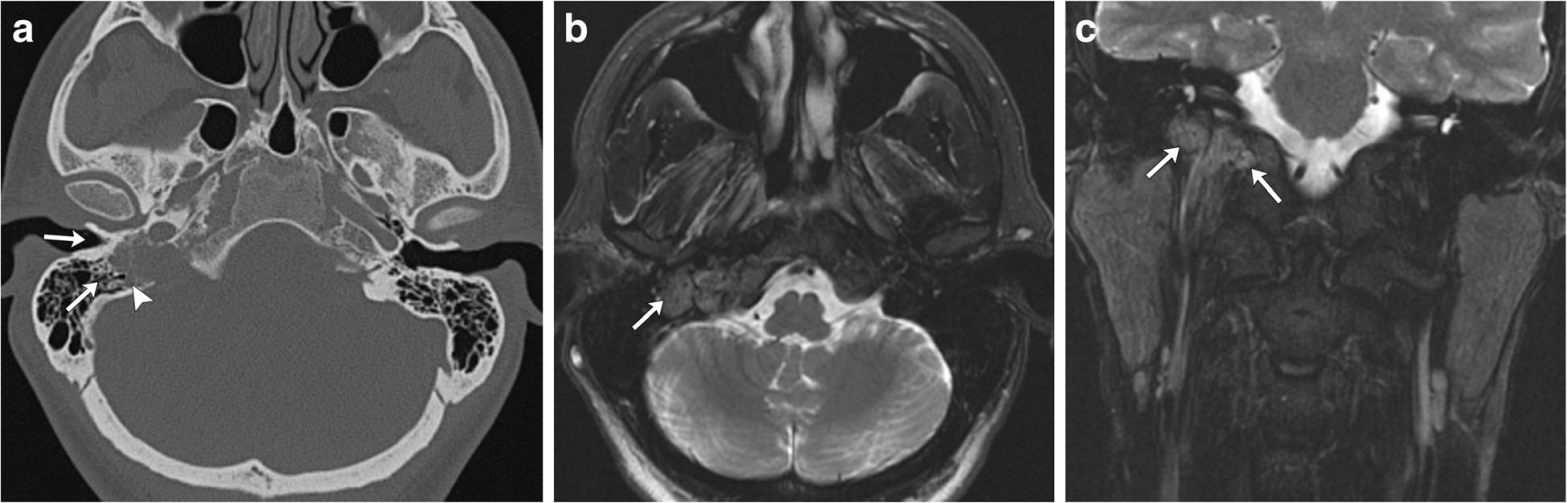
Axial CT (**a**) demonstrates erosion of the right jugular bulb, with a moth-eaten appearance of the walls of the right jugular foramen (white arrowhead). There is no invasion or extension into the middle ear, as the cochlear promontory remains intact (white arrows). Axial (**b**) and coronal (**c**) T2-weighted FSE MR images demonstrate a well-defined, predominantly T2-hyperintense, expansile lesion centered in the right jugular foramen (white arrows). Characteristic “salt and pepper” appearance is present, with punctate regions of hyperintensity representing the “salt,” while small flow voids represent the “pepper.” No extension into the middle ear is seen

These tumors expand from the jugular foramen along the path of least resistance and commonly extend into the pneumatized portions of the temporal bone, adjacent vascular and neural foramina, and eustachian tube. Characteristically, jugular paragangliomas expand aggressively, with later stages resulting in a “moth-eaten” appearance of the temporal bone due to associated bony erosion (Fig. 9). This distinguishes jugular paragangliomas from tympanic paragangliomas, which are typically smaller and less aggressive, sparing the jugular bulb and ossicles. Because of the frequency of osseous involvement, dedicated temporal bone CT is essential for preoperative evaluation of jugular paragangliomas. Any dehiscence of the floor of the tympanic cavity or extension into the tympanum should be excluded. Additionally, when evaluating for jugular paragangliomas, care must be made to exclude an aberrant course of the internal carotid artery, as injury during resection can result in significant morbidity. Evaluation for an absent or hypoplastic vertical segment of the carotid canal can reliably exclude this normal variant. MR provides a complementary role in evaluation, as it is more sensitive for any associated intracranial extension, which if present can significantly alter management [16].

### Tympanic paragangliomas

Tympanic paragangliomas are the most common tumor of the middle ear [23]. They arise from the tympanic branch of the glossopharyngeal nerve (CN IX), also known as the Jacobsen nerve. The mean age of presentation is 60 years of age, and there is a marked female predilection, with 80–90% of tympanic paragangliomas seen in women [6, 23]. Like jugular paragangliomas, they most commonly present with pulsatile tinnitus or unilateral hearing loss. These tumors are commonly seen as red or blueish, pulsatile masses within the middle ear on otoscopic examination [23].

**Fig. 10:**
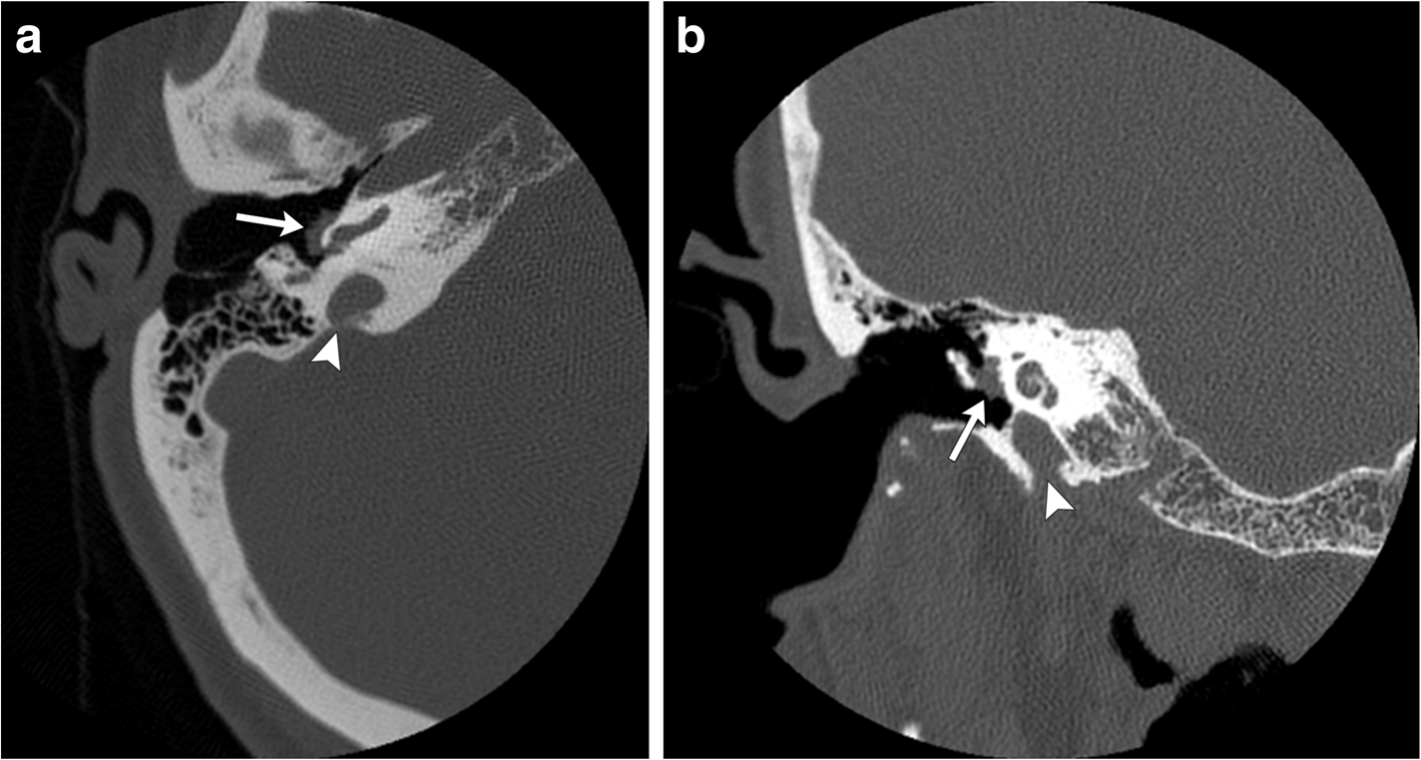
Axial (**a**) and coronal (**b**) CT images through the right temporal bone demonstrate a smooth, well-defined smooth soft tissue mass arising from the lateral aspect of the cochlear promontory, expanding outward into the middle ear (white arrows). Note the intact jugular bulb (white arrowhead)

The imaging modality of choice for evaluation of tympanic paragangliomas is high-resolution contrast-enhanced temporal bone CT. These tumors present as a soft tissue mass arising from the cochlear promontory, confined to the tympanic cavity (Fig. 10). Typically, the ossicles are spared, and osseous involvement is uncommon. Any osseous erosion around the jugular bulb must be excluded, as this is considered diagnostic of a jugular or jugulotympanic rather than tympanic paragangliomas. Tympanic paragangliomas are highly vascular tumors and demonstrate marked enhancement on both CT and MR, which differentiates these lesions from other common tympanic tumors such as cholesteatomas. MR is useful for further characterization of the soft tissue component of the mass and can help to exclude mimics such as inspissated secretions.

### Jugulotympanic paragangliomas

**Fig. 11:**
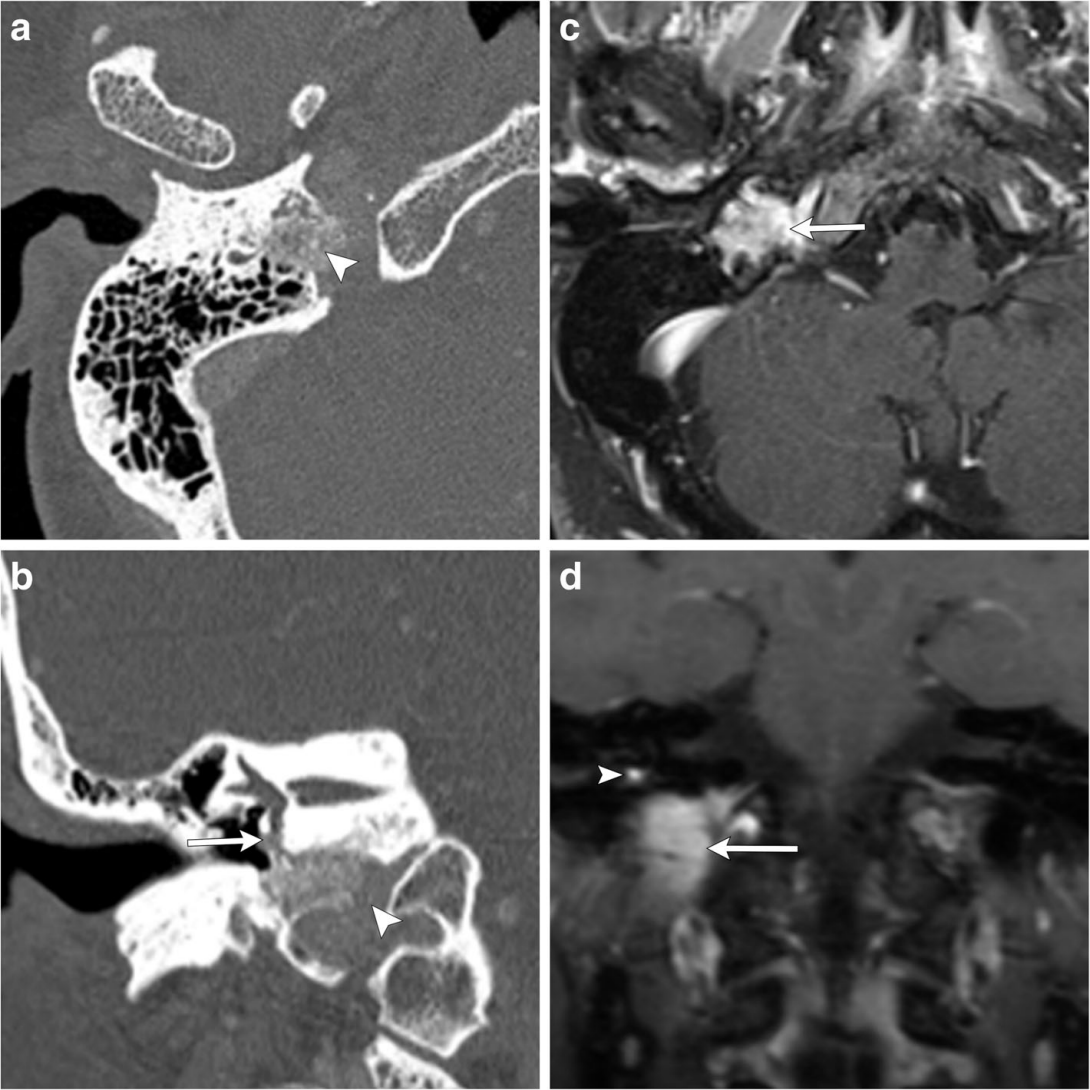
Axial (**a**) and coronal (**b**) CT images demonstrate erosion of the right jugular bulb, with a moth-eaten appearance of the walls of the right jugular foramen (white arrowheads). This destructive lesion expands into the middle ear, with invasion of the cochlear promontory (white arrow). Axial (**c**) and coronal (**d**) T1-weighted post-contrast MR images demonstrate avid enhancement of the mass which arises between the jugular foramen (white arrow) and the cochlear promontory (white arrowhead). The mass fills the jugular foramen and extends into the middle ear anterolaterally

Jugulotympanic paragangliomas are defined as paragangliomas which cannot be definitively classified as jugular or tympanic in origin. Their defining characteristic is invasion of both the jugular foramen and middle ear, without clearly arising from either of these spaces. Like their jugular and tympanic counterparts, the most common presenting symptoms include pulsatile tinnitus; however, due to their large size, deficits of cranial nerves IX and X are frequently seen. Temporal bone CT and MR are both utilized to evaluate for the epicenter of the lesion and to evaluate for erosion of the jugular bulb and extension into the jugular foramen and middle ear (Fig. 11).

## Conclusion

Paragangliomas are rare head and neck tumors but are an important differential consideration when a mass is seen in their characteristic locations. They commonly occur within the carotid space, jugular foramen, middle ear, and along the course of the vagus nerve. These vascular tumors demonstrate avid enhancement on CT and MR and often exhibit a characteristic “salt and pepper” appearance. Knowledge of their common locations and characteristic appearance on imaging can allow the radiologist to promptly and accurately diagnose these generally benign lesions. Additionally, by understanding the important imaging findings and associated anatomic involvement of each type of paraganglioma and concisely conveying them to referring clinicians and surgeons, the radiologist can impact subsequent management decisions for the patient.
